# Paramagnetism in Microwave-Synthesized Metal-Free Nitrogen-Doped Graphene Quantum Dots

**DOI:** 10.3390/ma16093410

**Published:** 2023-04-27

**Authors:** Flavia P. N. Inbanathan, Katherine Leslee A. Cimatu, David C. Ingram, Uriel Joseph Erasquin, Kiran Dasari, Muhammad Shehzad Sultan, Muhammad Sajjad, Vladimir Makarov, Brad R. Weiner, Gerardo Morell, Payman Sharifi Abdar, Wojciech M. Jadwisienczak

**Affiliations:** 1 School of Electrical Engineering and Computer Science, Ohio University, Athens, OH 45701, USA; 2 Department of Chemistry and Biochemistry, Ohio University, Athens, OH 45701, USA; 3 Department of Physics and Astronomy, Ohio University, Athens, OH 45701, USA; 4 Department of Physics, University of Puerto Rico—Rio Piedras Campus, San Juan, PR 00925-2537, USA; 5 Molecular Sciences Research Center, University of Puerto Rico, San Juan, PR 00926-2614, USA; 6 Department of Chemistry, University of Puerto Rico-Rio Piedras Campus, San Juan, PR 00925-2537, USA; 7 Department of Chemical and Biomedical Engineering, Institute for Corrosion and Multiphase Flow Technology, Ohio University, Athens, OH 45701, USA

**Keywords:** NGQDs, microwave synthesis, UV-Vis, fluorescence, AFM, TEM, XPS, VSM

## Abstract

Nitrogen-doped graphene quantum dots (NGQDs) have gained significant attention due to their various physical and chemical properties; however, there is a gap in the study of NGQDs’ magnetic properties. This work adds to the efforts of bridging the gap by demonstrating the room temperature paramagnetism in GQDs doped with Nitrogen up to 3.26 at.%. The focus of this experimental work was to confirm the paramagnetic behavior of metal free NGQDs resulting from the pyridinic N configuration in the GQDs host. Metal-free nitrogen-doped NGQDs were synthesized using glucose and liquid ammonia as precursors by microwave-assisted synthesis. This was followed by dialysis filtration. The morphology, optical, and magnetic properties of the synthesized NGQDs were characterized carefully through atomic force microscopy (AFM), transmission electron microscopy (TEM)), UV-VIS spectroscopy, fluorescence, X-ray photon spectroscopy (XPS), and vibrating sample magnetometer (VSM). The high-resolution TEM analysis of NGQDs showed that the NGQDs have a hexagonal crystalline structure with a lattice fringe of ~0.24 nm of (1120) graphene plane. The N1s peak using XPS was assigned to pyridinic, pyrrolic, graphitic, and oxygenated NGQDs. The magnetic study showed the room-temperature paramagnetic behavior of NGQDs with pyridinic N configuration, which was found to have a magnetization of 20.8 emu/g.

## 1. Introduction

Graphene quantum dots (GQDs), which are of single or multiple atomic layers with sizes being less than 10 nm, show remarkable thermal, electrical, and optical properties due to edge effects, impurities, and defect states that enable excitation-dependent luminescence behavior. It has been also shown that GQDs have advantages, such as good water solubility, low toxicity, and bio-compatibility [1,2,3,4]. GQDs have also been explored for applications in the fields of bio-imaging [5], sensors [6], catalysis [7], photovoltaic devices [8], and supercapacitors [9], among others.

The study of the magnetic properties of GQDs is of great interest wherein the observed magnetism is assigned to different factors [10], such as functionalization [11], presence of defects [12], morphology [13], edge states [13,14], and adatoms and substitutional hetero atoms [15,16,17]. In general, the magnetic properties of GQDs are governed by electron edge states, zero edge states (ZES), and dispersed edge states (DES), as demonstrated by tight-binding approximation calculations [18]. The partially hydroxylated and carboxylated GQDs exhibited a half-metallic state under the same electric-field intensity, whereas fully oxidized stable non-magnetic GQDs behave as spin-selective semiconductors when compared with the fully hydrogenated GQDs [19]. It was shown that graphene nanocrystals obtained by sonic exfoliation of graphite were strongly diamagnetic, similar to graphite. However, a weak paramagnetic behavior below 50 K, associated with zigzag edges, was also observed [20]. Furthermore, it was reported that paramagnetic behavior with the magnetization of 2.41 emu/g from hydroxylated graphene demonstrated that stable hydroxyl (−OH) groups induced magnetic moments on the basal plane of the graphene sheet [21]. Overall, these studies observed that most of the GQDs are either diamagnetic or weekly paramagnetic.

On the other hand, the doping of GQDs with light elements such as boron (B), fluorine (F), and nitrogen (N) has been reported in terms of photoluminescence and magnetism. Between boron and nitrogen, N has more similar atomic radii and chemical bond lengths to those of carbon, and it can be a more fitting dopant than other elements for inducing new enhanced optical, catalytic, sensing, and spintronic properties. GQDs exhibit paramagnetic properties at low temperatures of 2 K, while at the high temperature of 300 K, they exhibit diamagnetic properties, showing a switching behavior at 2 K and 300 K respectively. B-doped GQDs show strong paramagnetic behavior with a magnetization value of 2.935 emu/g at 6 K and 0.0398 emu/g at 300 K, respectively [22]. F-doped GQDs exhibit paramagnetism of 0.68 emu/g due to the preservation of magnetic edge states by F atoms [23]. Graphene behaves as a diamagnet, if the atomic concentration of nitrogen content is less than 5%; however, the doping of graphene with N can break the delocalized π-bonding network of graphene lattice that can induce magnetic moments [24]. N-doped graphene of less than 5.1 at.% of graphitic nitrogen was found to be non-magnetic, whereas for more than 5.1 at.% of graphitic nitrogen, it showed a ferromagnetic state at ~69 K and displayed a saturation magnetization of 1.09 emu/g. However, the magnetism switched from ferro- to paramagnetic when the temperature increased above 70 K [25]. In addition, the presence of chemisorbed nitrogen can also be a factor in lowering the effect of imprinting the magnetism into GQD [25].

On the other hand, NGQDs have been of research interest primarily because of the tunability of their optoelectronic properties due to the concentration of N-doping state in the hexagonal graphene lattice structure [25,26]. The presence of N in a graphene lattice structure has been classified into pyridinic, pyrrolic, graphitic, oxidized, and amine states [26]. The formation of the N-state in GQDs strongly depends on the selection of synthesis methods, precursors of carbon, and precursors of nitrogen doping in GQDs. The synthesis parameters, such as temperature, pH, and filtration techniques, were also found to affect whether high NGQDs yield is achieved [27]. XPS studies revealed that these N-states observed from the N1s spectrum deconvoluted into several individual peaks and were assigned to pyridinic N (398.1–399.3 eV), pyrrolic N (399.8–401.2 eV), quaternary N (401.1–402.7 eV), and oxidized N at ~402.8 eV. The literature shows that NGQDs have been synthesized using the electrochemical approach [28], hydrothermal treatment [29], chemical vapor deposition [30], pulsed laser deposition [31], and microwave synthesis [32]. Among these techniques, the microwave synthesis method is a simple technique that offers fast and efficient processing of materials by a higher heating rate achieved through uniform and the concurrent thermal reaction of a material(s), through dielectric polarization [33]. It is a cost-effective method that offers high reproducibility, energy saving, and higher yield in a shorter synthesis time, along with numerous advantages, such as lower processing cost, particle size reduction, narrow particle size distribution, and high purity over other conventional approaches. The basic principle of the commercial microwave heating method is the direct interaction of microwaves at a frequency of 2.45 GHz with the charged particles of materials, resulting in the production of heat via collision or by conduction [34]. Numerous works have explored the magnetic properties of GQDs [10,11,12,13,14,15,16,17,18,19,20,21,22,23,24,25,26], but there are limited experimental reports available so far, confirming the magnetic properties can arise out of the N doping in GQDs. Hence, this work focuses on the demonstration of magnetism in metal-free NGQDs due to the presence of pyridinic N-configuration in GQDs resulting in the observation of paramagnetism. The results presented here provide evidence that the paramagnetic behavior is only due to the presence of nitrogen doping and not due to the presence of ex situ metallic species typically used as catalysts for synthesizing GQDs [17,21]. The subsequent sections present the microwave synthesis method of metal-free N-doped GQDs (NGQDs) and characterization studies of NGQDs by studying their optical, structural, and magnetic properties.

## 2. Materials and Methods

### 2.1. Synthesis Procedure

In total, 200 mL of deionized water was added to 40 g of glucose (Sigma Aldrich, ≥99.5%) and 20 mL of liquid ammonia and stirred well. The mixture in a polypropylene container was then heated for 1 min in a commercial microwave oven at 900 W. The resultant solution was stirred for an hour at 50 °C. Then, the solution was dialyzed in deionized water using a 1 kDa dialysis tubing (Spectra/Por 6 Dialysis membrane of 1 kDa MWCO) until the pH of the outside solution dropped to 6.5 from 11. Then, the excess water was evaporated using a vacuum chamber, by applying heat not exceeding 50 °C. The product was a deep brown colored concentrated gel-like substance. In order to extract the NGQD powder, the decanting process was adopted. For the decanting process, the NGQD yield was taken in a 40 mL vial and filled with cold ethanol as solvent, ultrasonicated for 50 min, and allowed to sit for 8 h. The next day, the solvent above the yield was removed and the decanting procedure was repeated until the solvent became clear. The resultant sample had a pale-yellow color. The remaining solvent, if any, was removed using a rotary evaporator. The yield of the NGQDs was 2.691 g before decanting and 0.9716 g after the decanting process. In our microwave synthesis method, with 1 min of exposure, metal-free graphene quantum dots with N doping were formed, especially with a majority of pyridinic N configuration.

### 2.2. Characterization Methods

The optical properties of NGQDs were studied with a UV-Vis spectrometer (Agilent HP-8453 diode-array spectrophotometer, CA, USA) and a fluorometer (PTI FelixGx, Horiba, Piscataway, NJ, USA). The morphology was studied using atomic force microscopy (AFM, Asylum Research MFP-3D-SA, CA, USA) and transmission electron microscope (HRTEM JEM 2100F High-Resolution Transmission Electron Microscope, MA, USA). The elemental composition analysis was performed using X-ray Photoelectron Spectroscopy (XPS, Kratos XSAM 800 electron spectrometer with a 1484 kα Al X-ray source utilizing 120 W power and pass energy of 40 eV, NY, USA). The vibrating-sample magnetometer (VSM, Lake Shore 736, OH, USA) was employed to measure the field-dependent magnetization (*M–H*) of NGQDs at 300 K temperature. The magnetic field of VSM was adjusted in the range of (1.5 kOe ≤ H ≤ 1.5 kOe) to measure the magnetization (*M*) versus magnetic field (*H*).

## 3. Results and Discussion

### 3.1. Morphology

**Figure 1:**
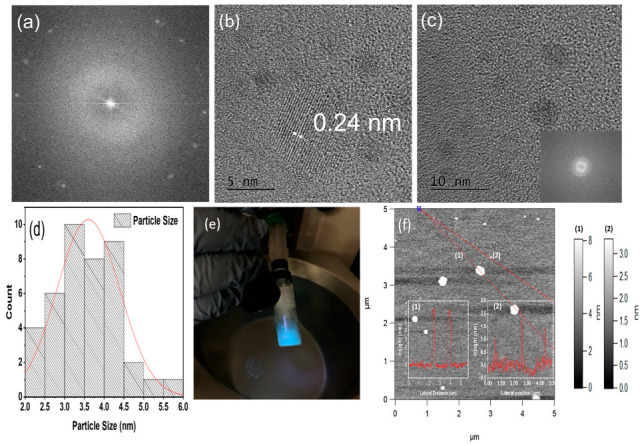
(**a**) SAED pattern, (**b**) TEM image of NGQDs at 5 nm, (**c**) TEM image of NGQDs at 10 nm, (**d**) histogram of NGQDs particle size distribution from TEM scan, (**e**) synthesized NGQDs under UV light exposure, and (**f**) AFM image of NGQDs with different line profiles (insets numbered (1) and (2)), showing the corresponding height of the selected particles.

Commercial microwave ovens use microwaves with a frequency of 2.45 GHz with a wavelength of 12.5 cm and are significant to induce nucleation or growth of material. Generally, microwaves interact with polar molecules. This interaction leads to the rotation and vibrational motion of polar molecules and generates heat within the material [35]. The precursors used in our synthesis method are polar molecules that result in uniform heating of the material. The microwave radiation over the polar molecules of glucose, liquid ammonia, and water resulted in a strong and quick interaction, resulting in graphene quantum dots doped with nitrogen that justifies the fact microwave synthesis creates significant structural modifications, very quickly. The obtained NGQDs were investigated for their size distribution and crystalline nature using TEM and AFM. Figure 1a–c shows the TEM image that confirms the NGQDs are nano-sized hexagonal particles, well-dispersed in the size ranging from 2 nm to 5.8 nm and with a lattice fringe of 0.24 nm (Figure 1b). The histogram fitted by the Gaussian curve shows an average diameter distribution of 3.5 ± 0.08 nm determined from a total of 44 particles, as shown in Figure 1d. Figure 1e depicts the turquoise blue fluorescence emission of the synthesized NGQDs under UV light exposure, verifying the dominant presence of NGQDs of an average size of 3.5 nm. Initially, 70 µL of 2.5% concentrated NGQDs was spin-coated using VTC-100 Vacuum Spin Coater on a clean mica substrate. The spin coating process involves spinning the sample at 2000 rpm for 10 s, then finally spinning the sample at 4000 rpm for 30 s. The first spinning action deposited the sample, while the second spinning action evaporated the solvent used. The AFM image of NGQDs with a scan size of 5 µm × 5 µm shown in Figure 1f reveals that the NGQDs’ topographical height is in the range between ~1 nm to ~6.8 nm. In particular, the larger particles (~6.8 nm) are due to the aggregation of small NGQDs, which were not effectively dispersed during the prolonged ultrasonication process (i.e., 50 to 100 min here) or NGQDs’ spontaneous agglomeration before a sample preparation for AFM studies. The AFM results confirm the size of the individual quantum dots obtained from the TEM analysis. 

### 3.2. Elemental Analysis

**Figure 2:**
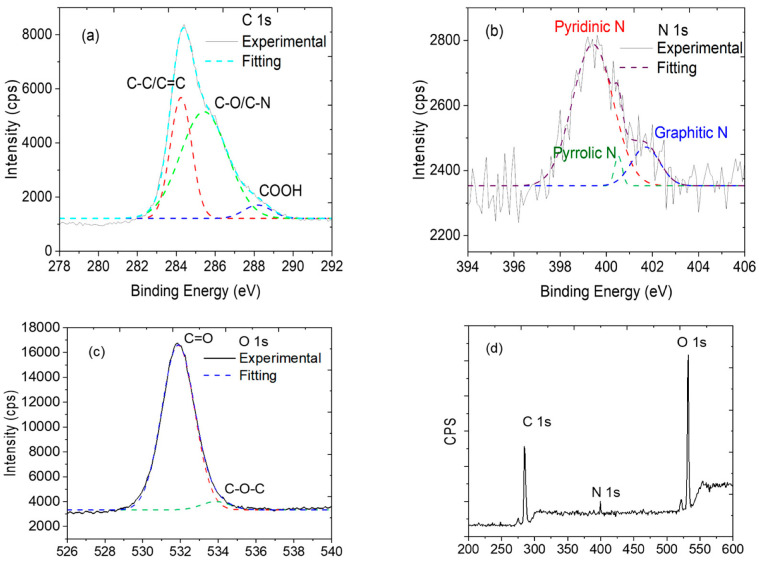
XPS spectra with the deconvoluted components of (**a**) C 1s, (**b**) N 1s, (**c**) O 1s, and (**d**) wide-energy-range XPS spectra.

The optical, electronic, and magnetic properties not only depend on doping concentration, but also depends on how the dopant atoms are bonded to the graphene atoms. This can be seen through the XPS. High-resolution XPS spectra of C1s, N1s, and O1s regions were deconvoluted and fitted using Sherley and Gaussian–Lorentzian (GL) fitting equations, as shown in Figure 2. The corresponding fitted curves that are obtained from CASA XPS are shown as dotted and colored lines in the spectra. The regions of all the elements are subtracted using Shirley’s background and are fitted with the GL (30) function, which is a standard for graphene-based samples.

**Figure 3:**
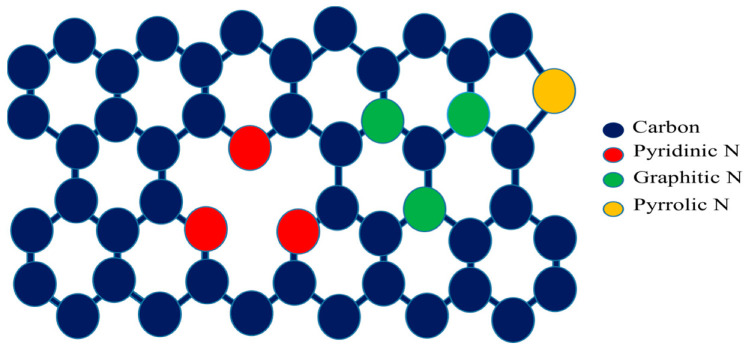
Illustration of the structure of N-doped GQDs.

The XPS results show ~3.26% of nitrogen doping in NGQDs at the atomic surface level. The three peaks positioned at 284.5, 399.5, and 532.5 eV were assigned to the binding energies of C 1s, N 1s, and O 1s, respectively. As can be seen in Figure 3, the C 1s peak has three sub-peaks, C-C/C=C (observed at 284.34 ± 0.02 eV), C-O/C-N (at 285.42 ± 0.07 eV), and COOH (at 288.18 ± 0.15 eV), which matches with the bond combinations as observed in the UV-vis experiment. From the XPS analysis, it was noted that 64% of the total C-atoms are participating in C=C bonding, whereas 16% of total carbons are active in bonding with oxygen and around 20% of the carbon atoms are found to be bonded to nitrogen. The N 1s peaks corresponding to pyridinic N, pyrrolic N, and graphitic N were observed at 399.4 ± 0.07, 400.5 ± 0.08, and 401.7 ± 0.17 eV respectively, and the deconvoluted spectra are provided in Figure 2a–c along with a wide energy range XPS spectrum in Figure 2d. Out of the 20% nitrogen, bonding with C, we observed that 90 ± 1% of the nitrogen atoms are bonded to carbon (predominantly pyridinic, moderate graphitic, and minimum pyrrolic nitrogen), and the remaining are bonded with oxygen and amine. In addition, the O 1s peaks at 531.8 ±0.02 eV and 533.86 ± 0.2 eV are associated with oxygenated functional groups C=O and C-O-C respectively. Both nitrogenated and oxygenated functional groups contribute to the good solubility of NGQDs in water. As shown in Figure 3, the NGQDs can be widely seen as three different configurations such as graphitic N, a simple substitutional bonding, where three carbon atoms are substituted by three nitrogen atoms in a six-membered graphene structure; pyridinic N, where N-atoms bond with carbon atoms in graphene lattice structure along with vacancy in the nearby C-N bonds; pyrrolic N, where N-atoms bonds with carbon in a five-membered graphene lattice with a defect. N doping in GQDs can exist in zigzag and armchair edges.

The properties of NGQDs depend on shape, size, and the presence of N-atoms in the GQDs, their bonding with neighboring atoms, and their stability to exist in the N-configurations, as shown in Figure 2b and Figure 3. In this work, the synthesis was carried out at a temperature not exceeding 50 °C at any stage of synthesis. This temperature plays an important role in having more NGQD yield with pyridinic N (399.4 eV) than the pyrrolic N (400.5 eV) and graphitic N (401.7 eV). As reported in [34], lower processing temperatures resulted in pyridinic N, and higher processing temperatures yielded graphitic N-dominant NGQDs. In our work, the synthesis temperature did not exceed 50 °C, resulting in pyridinic N, thus validating the importance of lower temperatures during the growth process of the NGQDs. The N dopants in GQDs were reported to be stable, while the N-substitution is on the edge of the atomic structure. Pyridinic N can result in N-type or P-type NGQD, based on whether the N dopant is bonded to hydrogen or not. Pyridinic N could exist in the form of N, N_2_, or N_3_, and Pyridinic N_3_, as reported in [36], and exhibited paramagnetism.

### 3.3. Optical Properties

**Figure 4:**
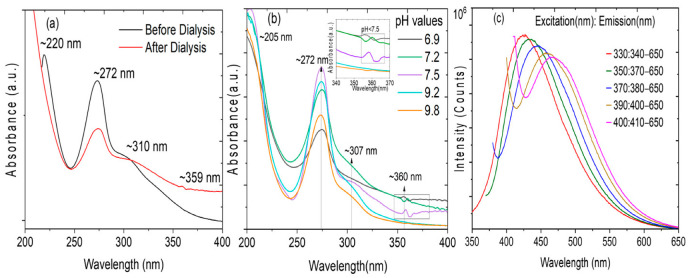
(**a**) Absorbance spectra of NGQDs before and after dialysis, (**b**) absorbance spectra of NGQDs at different pH values, and (**c**) fluorescence spectra of NGQDs at different excitation wavelengths.

The synthesized NGQDs were analyzed for their optical properties. The absorption spectra of NGQDs are shown in Figure 4a, with a peak positioned at ~272 nm, assigned to the *π* − π* and *n* − π* transitions of aromatic *sp*^2^ carbon and C-N bonds [32,37,38,39]. This shows the strong electron affinity of nitrogen atoms in the domain of graphene quantum dots [34,37]. Three absorption peaks at 220 nm, 272 nm, and 310 nm were seen before dialysis and four peaks were seen at 210 nm, 272 nm, 307 nm, and 360 nm after dialysis. The peaks at 210 nm and 220 nm correspond to C-C bonds. The peaks at ~307 nm corresponded to C=O [32,38]. The minute peaks at ~359 nm correspond to C=O/C=N, respectively, as a result of the non-bonding orbitals from pyridinic N [37,40].

As seen in Figure 4a, the maximum intensity of absorbance peaks decreased after dialysis, since the nanoparticles have been narrowed down by their size by the 1 kDa dialysis membrane. The absorbance spectra measured at different pH values, as shown in Figure 4b, revealed that the peak observed at ~360 nm evolved when the pH value < 7.5. The peak evolution can be attributed to the formation of C=N/C=O functional groups due to *n* − π* electronic transitions in the NGQDs [40]. Figure 4c shows the excitation-based emission fluorescence (FL) spectra with different intensity peaks, recorded for different excitation wavelengths from 300 to 400 nm. The FL spectra reveal the inhomogeneity of NGQDs with the presence of different sizes of quantum dots as measured using TEM. The emission peaks of FL spectra of NGQDs exhibit a shift towards longer wavelength with a further increase in excitation wavelength. This is associated with the presence of quantum dots of different sizes ranging from 2 nm to 5.8 nm. However, the emission peak with the highest intensity is associated with the NGQDs of size ~3.5 nm, as verified through the color of fluorescence emission, and the quantum dot size measurement using TEM analysis, as shown in Figure 1d. The decrease in peak intensity along with a shift in emission spectra reveals that the presence of a fraction of NGQDs of other sizes, as shown in the TEM histogram, and AFM is lesser in concentration compared to the predominant 3.5 nm size. Apart from this particle size effect, the doping effect and increase in carbon-nitrogen/carbon-oxygen functional groups in the graphene lattice structure, change in the bandgap due to the photo-oxidation, and HOMO to LUMO transition are among the other significant contributing factors for FL peak shift and improvement in the stability of the quantum dots [39,40,41,42]. With an increase in the excitation wavelength, the emission color of FL spectra shifts towards the longer wavelength, with emission peaks shifting from strong blue to a cyan color confirming the presence of NGQDs, as the GQDs only emit fluorescence in the blue region. The FL spectra show the strongest peak at approximately 430 nm (2.88 eV), excited by a wavelength of 330 nm (3.76 eV).

**Figure 5:**
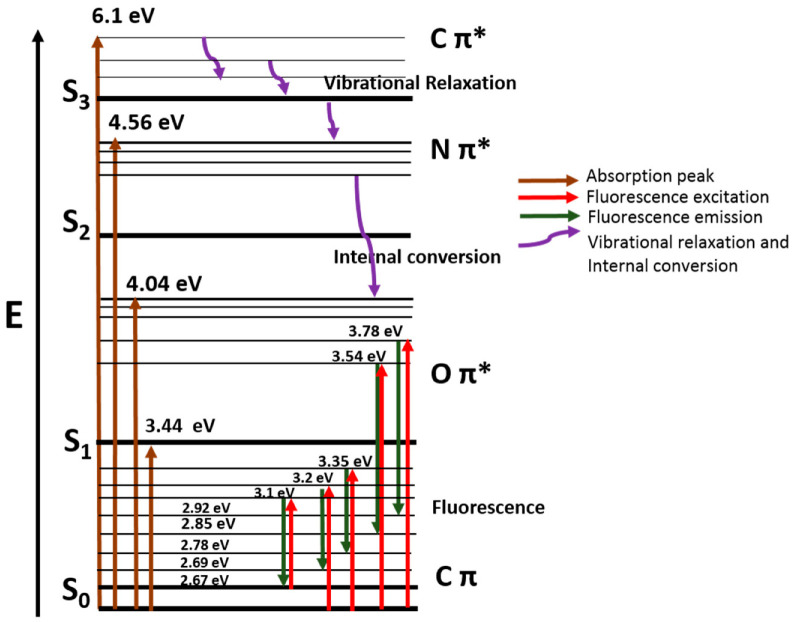
Jablonski diagram detailing the energy level structure of NGQDs from UV-Vis and fluorescence spectra, where π and π* represents low and high energy level orbitals, respectively.

A Jablonski diagram, as shown in Figure 5, has been created from the light absorption peaks and fluorescence excitation energy and emission peaks. This helps us to understand how NGQDs behave upon excitation and moves to states of higher energy before it returns to the ground state. *S*_0_, *S*_1_, *S*_2_, and *S*_3_ are the electronic energy states, and the different vibrational energies are shown using thick lines. Upon photoexcitation, the excited electrons shift from Cπ → Cπ* (6.1 eV), Cπ → Nπ* (4.56 eV), and Cπ → Oπ* (4.04 eV), then undergoes vibrational relaxation and internal conversion (purple distorted line) before entering fluorescence emission spectral energy level from 2.92 eV to 2.67 eV for the corresponding excitation energy levels from 3.78 eV to 3.1 eV, and finally returns to the ground state. The FL emission energy of Oπ* is from 3.78 to 2.92 eV and 3.54 eV to 3.2 eV.

### 3.4. Magnetic Properties

**Figure 6:**
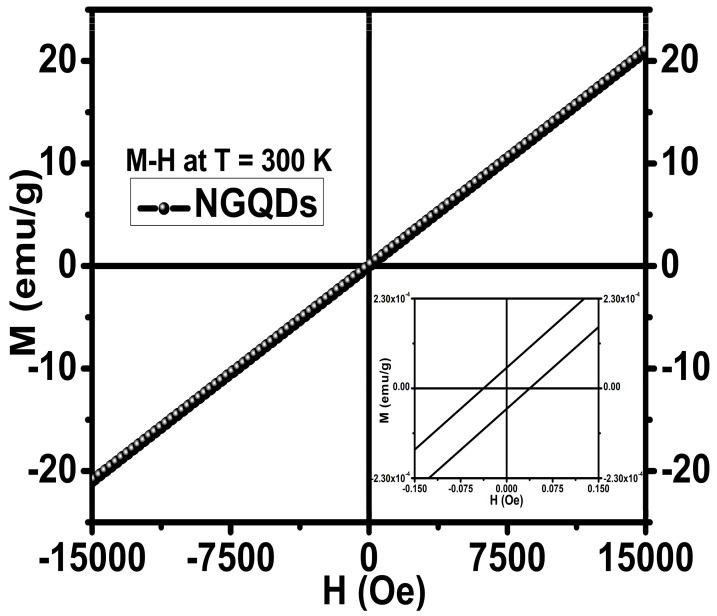
The magnetization *M–H* curves of NGQDs at 300 K with the PPMS system, employing the VSM module. The inset depicts the *M–H* curves in the low magnetic field region, showing negligible remanence (*M_R_*) and coercivity (*H_C_*).

Due to increasing interest in the magnetic properties of doped graphene quantum dots, the magnetic effects of nitrogen doping on graphene quantum dots were investigated using VSM with results shown in Figure 6. The presence of 3.26 at.% of firmly bonded nitrogen doping at the edges and defect sites in the graphene lattice structure of the synthesized NGQDs induced paramagnetic centers, arising out of unpaired conduction electrons in the orbitals, resulted in long-range magnetic ordering at 300 K. The surface defects that occur during the nitrogen doping via the chemical synthesis route allow the presence of functional groups of N, NH_2_, O, and OH in the graphene lattice, and this work focuses on analyzing the N doping in GQD. The synthesized NGQDs with predominantly present pyridinic N resulted in a paramagnetism with a magnetization of ~20.8 emu/g, being the notable and significant value among the other reported values from the literature, as presented here for a comparison, 0.680 emu/g (T = 2 K) [21], 2.41 emu/g [22], 2.935 emu/g (T = 6 K), 0.0398 emu/g (T = 300 K) [23], and 0.37 emu/g (T = 70 K) [25], for various doped graphene-based systems with OH, B, F, and N, respectively. It has been reported in [25] that the presence of graphitic N induces ferromagnetism at 69 K and the hysteresis shifted from ferromagnetism (0.65 emu/g) to paramagnetism (0.37 emu/g) at ~70 K. Based on our experimental study, we found that the microwave synthesis of NGQDs not only resulted in high yield of metal-free NGQDs, but also resulted in NGQDs that exhibited a high magnetization of 20.8 emu/g at 300 K. The linear shape of the magnetization curve reveals that the NGQDs have transverse anisotropy. The dominant mechanism behind the paramagnetism is due to the reversible rotation of magnetization toward the field direction that happens in the unpaired electrons from the pyridinic-rich NGQDs.

The quantum magnetic moments ‘*m*’ associated with possible spin can exist only in two possible forms, i.e., +*m* or −*m*. The higher magnetization value of ~20.8 emu/g indicates that the thermal agitation among the quantum dots is less at room temperature and, with the application of an external magnetic field, the magnetic moments are aligned easily, resulting in the paramagnetism of NGQDs. Figure 6 inset shows small values of remanence and coercivity around 0 Oe magnetic fields, confirming that the material is paramagnetic. The gap between the forward and reverse curves is very small, which is typical for paramagnetic materials and corroborates well with our conclusion that the studied NGQDs are paramagnetic. Furthermore, an increase in magnetism can be achieved by targeting the oxygen reduction reaction with the presence of more pyridinic N configuration than the pyrrolic and graphitic N in the edges [43]. The cited references show much smaller emu/g, whereas our results show 1–2 orders of larger magnitude value. This paramagnetism can be attributed to the predominantly present pyridinic N configuration in the sample. Pyridinic N doping in GQD happens at the edge of the GQDs along with a vacancy, and hence, there is a hole to accept atoms. The graphitic N structure happens by substitution but without a vacancy, meaning the substitutional nitrogen atoms are bonded to carbon atoms in the carbon structure. This shows that the graphitic N may not be the source behind the paramagnetism seen in the sample. This has been validated by the results reported in [34], through density functional theory computation. Yutomo et al. have shown that pyridinic N resulted in p-type metallic conductivity and paramagnetism with the increase in magnetic moments with the doping concentration of pyridinic N, and graphitic N with n-type conductivity and non-magnetism, thereby eliminating the role of graphitic N in inducing magnetism [37]. The insignificant presence of the pyrrolic N concentration in the material may not play a role in inducing magnetism. Hence, the paramagnetism appears to be due to pyridinic N and not due to graphitic and pyrrolic. Furthermore, we postulate that the NGQDs magnetic susceptibility could be increased with an increase in the nitrogen doping concentration. Therefore, one may expect that the further evolution and enhancement of the magnetically active nitrogen configurations in NGQDs can be achieved. Hence, increasing nitrogen concentration in NGQDs by, e.g., synthesizing material using a non-equilibrium synthesis method, can result in the observation of magnetic ordering leading to, e.g., superparamagnetism (not shown here [44]). As discussed earlier, Blonski et al. have demonstrated that graphene doped with nitrogen below 5 at.% is non-magnetic but exhibited a ferromagnetic state with a saturation magnetization at ~70 K when the doping level exceeds this nitrogen content [25]. We believe that our magnetic characterization results corroborate that prediction. The actual N doping level in the GQDs studied here was 3.26 at.%, and it was smaller than the predicted N concentration required for inducing magnetic ordering. Simply, N doping at such levels did not imprint a specific and dominant magnetic configuration because the induced paramagnetic centers were far from each other, precluding the establishment of a long-range magnetic ordering. Additionally, one can notice that the isothermal coercivity in our case is in the range of 10^−4^ (emu/g) at 300 K, indicating that the overall magnetic characteristics of the tested N-doped GQDs are overwhelmingly paramagnetic at 300 K, even with some clue that there might be other minor magnetic contributions. Furthermore, synthesis of N-doped GQDs with an N content above ~5 at.% will require confirming unambiguously the transition from the paramagnetic to the superparamagnetic or ferromagnetic regime on lowering the temperature [44]. However, relying on the current experimental observations the studied NGQDs are paramagnetic at 300 K. Therefore, with our present findings and magnetic properties arising out of NGQDs, we believe that future research on this topic will pave a new direction in energy and bio-medical applications [17] and bio-imaging [22,34,45], where metal-free magnetic NGQDs are desirable because of bio-compatibility or other constraints. The demonstrated magnetic properties also suggest an avenue to explore NGQDs for their magnetic functionalities in fluorescent NGQDs ink applications [46,47] and biosensors [48,49].

## 4. Conclusions

Metal-free NGQDS with an average particle size of 3.5 nm were synthesized from glucose and liquid ammonia precursors using microwave-assisted synthesis. Topographical studies revealed that the particle size has a narrow distribution. The optical studies showed that the NGQDs have a strong shift towards the longer wavelength with cyan color emission due to the electronic transition from HOMO to LUMO and compression in the bandgap. Elemental composition analysis showed the presence of more pyridinic N, along with less of the visible presence of graphitic, and almost negligible presence of pyrrolic and chemisorbed nitrogen. The presence of ~3.26 at.% nitrogen at a temperature of 300 K in the edges of the graphene structure seems to have induced the paramagnetic response in NGQDs. The experimental results show that NGQDs synthesized using microwave power resulted in NGQDS with a higher yield, photoluminescence with a redshift, and a paramagnetic response with a magnetic saturation of 20.8 emu/g at 300 K. This experimental work also confirms that the lower synthesizing temperature results in a pyridinic N-configuration in the graphene quantum dot structure, and that the pyridinic NGQDs makes the graphene material a potential candidate for magnetic and spintronic applications. Moreover, the microwave-assisted synthesis method paves the way for the cost-effective upscaling of metal-free quantum dots.

## Data Availability

For the data support, please contact the corresponding authors.
